# Tasks as needs: reframing the paradigm of clinical natural language processing research for real-world decision support

**DOI:** 10.1093/jamia/ocac121

**Published:** 2022-07-14

**Authors:** Asher Lederman, Reeva Lederman, Karin Verspoor

**Affiliations:** Faculty of Engineering and IT, School of Computing and Information Systems, University of Melbourne, Melbourne, Australia; Faculty of Engineering and IT, School of Computing and Information Systems, University of Melbourne, Melbourne, Australia; STEM College, School of Computing Technologies, RMIT University, Melbourne, Australia

**Keywords:** artificial intelligence, natural language processing, clinical decision support, clinical judgment, intersectoral collaboration

## Abstract

Electronic medical records are increasingly used to store patient information in hospitals and other clinical settings. There has been a corresponding proliferation of clinical natural language processing (cNLP) systems aimed at using text data in these records to improve clinical decision-making, in comparison to manual clinician search and clinical judgment alone. However, these systems have delivered marginal practical utility and are rarely deployed into healthcare settings, leading to proposals for technical and structural improvements. In this paper, we argue that this reflects a violation of Friedman’s “Fundamental Theorem of Biomedical Informatics,” and that a deeper epistemological change must occur in the cNLP field, as a parallel step alongside any technical or structural improvements. We propose that researchers shift away from designing cNLP systems independent of clinical needs, in which cNLP tasks are ends in themselves—**“tasks as decisions”**—and toward systems that are directly guided by the needs of clinicians in realistic decision-making contexts—“**tasks as needs.”** A case study example illustrates the potential benefits of developing cNLP systems that are designed to more directly support clinical needs.

## BACKGROUND

Electronic Health Records (EHR) have been rapidly adopted by hospitals and health clinics worldwide, with the intent of storing and collating data to support clinicians in making decisions at the point of care.^1^ However, it can be difficult to extract knowledge from the multiple data formats in EHR, in particular from unstructured texts. Consequently, Natural Language Processing (NLP) has been deployed for automated extraction, decoding, and analysis of free-text EHR data. Since the 1960s, clinical NLP (cNLP) research has led to advances in areas such as clinical note summarization,^2^^,^^3^ identifying diagnoses,^4^^,^^5^ and adverse drug reactions.^6^^,^^7^ However, the cNLP field has experienced an ongoing lack of deployed NLP systems in healthcare settings,^1^^,^^8–12^ and this problem is arguably growing despite—or possibly because—the increasing sophistication of cNLP systems. It is important that we now work to address the factors preventing the translation of cNLP applications into real-world clinical contexts. In this paper, we survey the cNLP landscape to understand these factors and propose a way forward.

To date, several NLP researchers have explored the factors and circumstances surrounding this lack of cNLP system implementation. Researchers have focused on flaws from the perspective of deficiencies in data (eg, scalability, insufficient standardization),^13^^,^^14^ models (eg, overfitting, biases),^15–17^ study designs (eg, simplifying assumptions, evaluation limitations),^16^^,^^18^ and software usability.^19^ All these concerns remain valid and continue to be addressed through development of suitable cNLP frameworks, standards, public datasets, and so on. However, we propose that it is the epistemological constraints within the cNLP field that most heavily detract from the intended clinical need and ultimately, deployment into clinical settings.^12^^,^^20^

Some cNLP models have been successfully deployed, where they were tasked with simple goals that directly complement clinical decision-making.^21–24^ In those few cases of successful (clinically useful) NLP deployments, the models typically targeted the “low hanging fruit,” such as efficient disease detection from explicit mentions, or identifying a family history of a disease.^12^^,^^22^^,^^25^ However, there are more numerous examples of as-yet-unadopted NLP systems, often developed within the framework of clinical decision-support systems (CDSS)^8^ that attempt to provide clinical recommendations, to use limited data to make predictions about future patient behavior or prognoses, or to audit clinical workflows.^26–31^ In short, they aim to make clinical decisions automatically. This includes our own work on early prediction of diagnostic-related group classification for patients,^32^ which has yet to find practical application.

These cNLP decision systems are designed under the assumption that their advanced logical or predictive power and far greater access to available clinical information, as well as the capability to synthesize this information, should facilitate more effective decisions than clinicians can make alone. It can even be said that these systems are based on an even stronger assumption, that is, that they facilitate more effective decisions than clinicians—thereby pushing the clinician outside of the decision-making. This then directly violates Friedman’s “Fundamental Theorem of Biomedical Informatics,”^33^ which states that “A person working in partnership with an information resource is ‘better’ than that same person unassisted,” and from which it follows that tools that seek to be independently more effective than a person unassisted violate a core principle of informatics research.

A chief example of the failed adoption of cNLP-based systems is IBM’s Watson Health platform. After the platform’s announcement in 2011, IBM invested at least $5 billion into its AI healthcare initiatives and it announced over 50 partnerships with healthcare providers to develop new AI-enabled clinical tools.^20^ Yet, nearly the entirety of these projects failed to lead to any useful clinical outcomes or platform deployments, often at great cost to healthcare organizations in terms of time, effort, and funds.^20^ It is important to ask why this project failed, especially given the apparent large-scale access the organization had to clinicians. Contributing factors to this failure appear to be that the system was unable to provide information that was not already easily accessible to clinicians,^34^ as well as lack of interoperability with EHR systems.^35^ In short, the cNLP system did not satisfy the basic needs of the clinicians who would work with it; that is, they were not working in partnership.

A validating example of this problem appears in a case study of a failed AI-based clinical cognitive agent in a hospital in Germany.^36^ The authors find “the cognitive agent had been given medical cocompetence with physicians, which meant that the agent wasn’t just a support tool but an autonomous operator. For the physicians, this was a step too far.”

We propose that the way to improve this situation is for cNLP researchers and clinicians to align in answering the following question:

*How can clinical decision-making best be supported through clinical NLP?*

By addressing this question, we are implicitly asking about the nature of rational clinical decision-making.^37^ The question reveals a set of underlying epistemological misalignments between cNLP designers and medical clinicians. For instance, cNLP systems that aim to predict outcomes or provide recommendations may offer an unrealistic quantification of real-world uncertainty,^38^ or they may aim for fixed forms of utility (ie, pre-established, stable outcomes) when these do not reflect the dynamic, real-world outcomes of the task at hand.^16^ In contrast, clinicians use a variety of complex reasoning methods^39^ and heuristics^40^ adapted to complex, dynamic environments, and they may purposefully ignore nonsalient information when making predictions, understanding the importance of trade-offs where there are known uncertainties.^41^ We argue that a deeper alignment on the objective of supporting clinical decision-making and identification of the relevant implications for cNLP systems would reduce these epistemological differences and ease the barriers to cNLP system adoption in the context of clinical decision support.

## KEY DIFFERENCES BETWEEN NLP AND CLINICIAN-LED DECISION-MAKING

Over 70 years ago, Herbert Simon, a founder of the Carnegie School of business management, introduced a scientific approach to business decision-making. He developed the now well-established precept of “unbounded rationality,” which states that an “ideal” decision-maker gathers, assesses, and weighs all relevant information according to some criterion, to maximize the likelihood of achieving their goal(s).^42–44^ cNLP researchers such as those that developed IBM Watson appear to have similarly assumed that computers are better positioned than clinicians to achieve unbounded rationality and should (eventually) set the standard for medical decision-making quality.^20^ Such claims have already been made for diagnostic imaging applications.^45^

However, individual clinician interpretation of narrowly defined language understanding tasks—rather than ideal decisions—is typically used as the “gold standard” for cNLP system development. For instance, numerous cNLP systems address information extraction of specific patient attributes, such as a patient’s smoking status,^46^ cardiovascular risk factors^47^ or social determinants of health,^48^ or focus on normalization of clinical data such as medication prescription details.^49–51^ While the information targeted by these tasks clearly is relevant to clinical decision-making, the tasks themselves are nevertheless distant from higher-level clinical tasks such as estimating prognoses or selecting treatments. This then ultimately impedes effective translation of these systems into practical clinical use.

While cNLP researchers often motivate model development in terms of the potential clinical utility of the tools (ie, savings of time and cognitive savings, reduction in human error), this utility often only applies to a small, quantifiable, and simplified portion of the overall clinical problem and clinicians may not see benefits from the small gains obtained there. We are not suggesting that data extraction or standardization of clinical narratives is a practically futile endeavor, but that the research focus should aim to enhance clinical practice, rather than (only) solving narrowly defined NLP problems.^10^

Still our primary concern is not with “low hanging fruit” of diagnostic or simple information extraction tasks within cNLP, with all of their inherent limitations. Rather, the assumption of unbounded rationality is leading NLP (and other AI researchers)^52^^,^^53^ researchers too hastily toward the “high hanging fruit”: NLP used directly to draw clinical conclusions and make useful recommendations.^27^^,^^54–57^ In aiming high, these tools appear to be jumping right over the sweet spot of utility for clinical users.

## WHERE TO FROM HERE? A NEW EPISTEMOLOGICAL MODEL FOR CNLP

**Figure 1.**
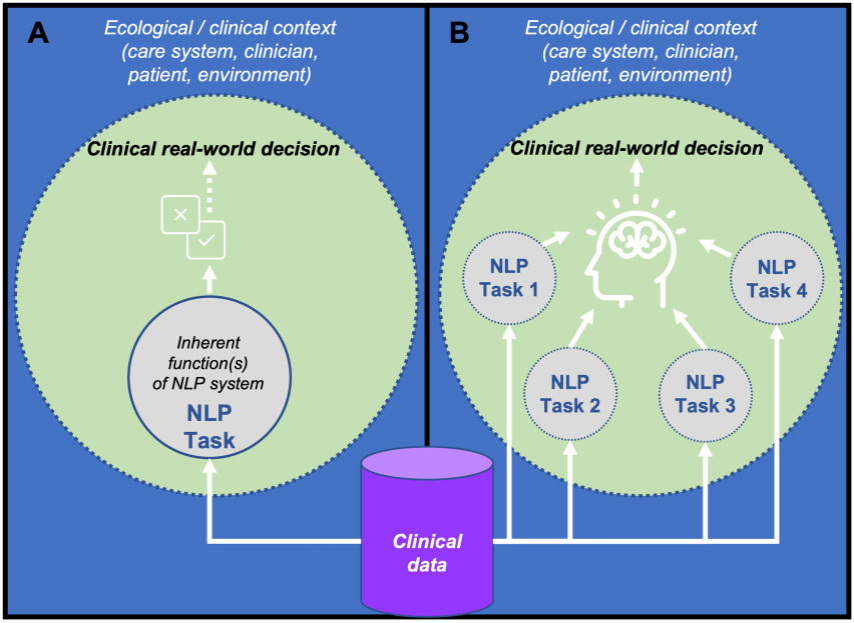
(**A**) Task as decision. The current model (deduced from the literature), where an NLP task (eg, classification, entity recognition) serves a discrete function that relates to a modeling objective, but is not explicitly designed to assist clinician decision-making. Dotted lines represent permeable boundaries; solid lines impermeable. (**B**) Task as need. The alternative model proposed by this paper, where one or more NLP tasks are directly designed to interact and contribute to providing evidence to support clinical decision-making. Dotted lines represent permeable boundaries.

We propose a more straightforward approach to address the barriers to deployment. Clinicians tend to use a “top-down” methodology to decision-making, where they fulfill the context-driven needs of relevant stakeholders using their reasoning, experience, heuristics, or protocols.^40^^,^^41^ Conversely, cNLP has generally taken a “bottom-up” approach due to inherently data-driven methods, where the system’s task is to optimally carry out predefined language analysis functions.^58^ In the current model (seen in Figure 1A), the NLP task is seen as an end in itself, often entirely distinct from clinician needs, and divorced from the wider clinical context. We refer to this model as the “**task as decision**” model, because NLP tasks and their evaluation remain intrinsically motivated, tied directly to a narrowly defined modeling objective, and not intended to work in concert with a clinician. An alternative model (captured in Figure 1B), the “**task as need**” model, implies that NLP tasks are designed to interact with, and directly address clinical needs in making decisions. Thus, assessing system utility by whether it answers a “what” question with strong reliability only leads to *inadvertent utility*. Rather, to ensure adoptability, researchers need to shift to addressing how reliably the system answers “why” and “how” questions.^59^

We take the use case of 2 systems that address modeling of hospital readmission of patients with cardiovascular disease^31^^,^^60^ as an example of a “task as decision” framing that can be reframed via the “task as need”, reflecting on what NLP tools would be needed to support the clinical decision of discharging a heart-failure patient. There are numerous cNLP works that have addressed hospital readmission that could be considered for this purpose^61^; the 2 works we focus on are transparent in defining the clinical concepts they target with NLP and therefore are arguably well-suited to this reframing.

Topaz et al^31^ analyzed clinical notes using a rule-based (regex-driven) NLP model. At the cohort level, they were able to successfully differentiate between re-admitted and nonreadmitted patients through an aggregated measure known as “ineffective self-management,” itself derived from the presence of terms such as “difficulties with outpatient adherence,” “excessive fluid intake,” and “skips medicines.” Navathe et al^60^ target several social risk factors by identifying terms related to drug abuse, housing instability, and poor social support in a patient’s clinical notes. Both systems are based on the Medical Text Extraction, Reasoning and Mapping System,^62^ utilizing dictionary-based term matching and context rules for disambiguation.

In their studies, the authors start with the factors extracted from the notes of patients, and use regression to model readmission outcomes based on these factors. While the identification of relevant patient factors is a seemingly valuable use of cNLP, and arguably in line with the “Tasks as Needs” framing, there are 2 concerns. First, the systems were not designed to fully address the real-world causes of re-admission in heart failure patients, or the full range of clinical factors relevant to making discharge decisions. A cursory review of the health services literature highlights the factors affecting hospital readmissions including the patient conditions these systems primarily target, such as congestive heart failure, chest pain, anxiety and depression, but also hospital operational factors such as nurse staffing care quality, staff responsiveness, length of stay, posthospital care coordination, and medication-related events.^63–67^ Many of these factors are entirely ignored by the cNLP systems, and arguably may not even be observable from a patient’s data, highlighting the need to design a system that allows a clinician to bring such factors to bear. Second, and critically, the systems are designed to produce a single number (eg, probability of readmission) or to make a recommendation (eg, safe to discharge) rather than explicitly surfacing and presenting the information that will support a clinician to make an informed decision within the broader context. They therefore exemplify the “Task as Decision” framing of cNLP, where the overarching aim of the system is to leverage text processing directly to make decisions.

Clinicians on the ward are likely to ask a number of “why” and “how” questions:

*“Why should I keep this patient in this ward bed, when there are four in the ER?”*^67^

*“How can I ensure this patient’s condition will be better at discharge than when she entered eight days ago?”*^63^^,^^68^

Clinicians might use heuristics to weigh up and compare the factors above (patient’s physical state and attitude, length of stay, family support, likely postdischarge care) when answering these questions.^40^^,^^41^ Using these heuristics, they can ignore the inherent uncertainty created by the idiosyncrasies of the individual patient and the complex, changing hospital environment.^41^^,^^69^

Therefore, we suggest that the cNLP tools should focus on those evidence-based factors, deemed relevant by clinicians, to *assist* the clinical reasoning rather than replace it. This requires both a cross-disciplinary research focus and stronger collaboration with clinicians in codesign of systems. The use of a cNLP system to detect mental health, attitudinal, or social variables deemed relevant by clinicians—and explicitly presented to them—would better support clinicians to consider the questions that they may ask at discharge.

**Figure 2.**
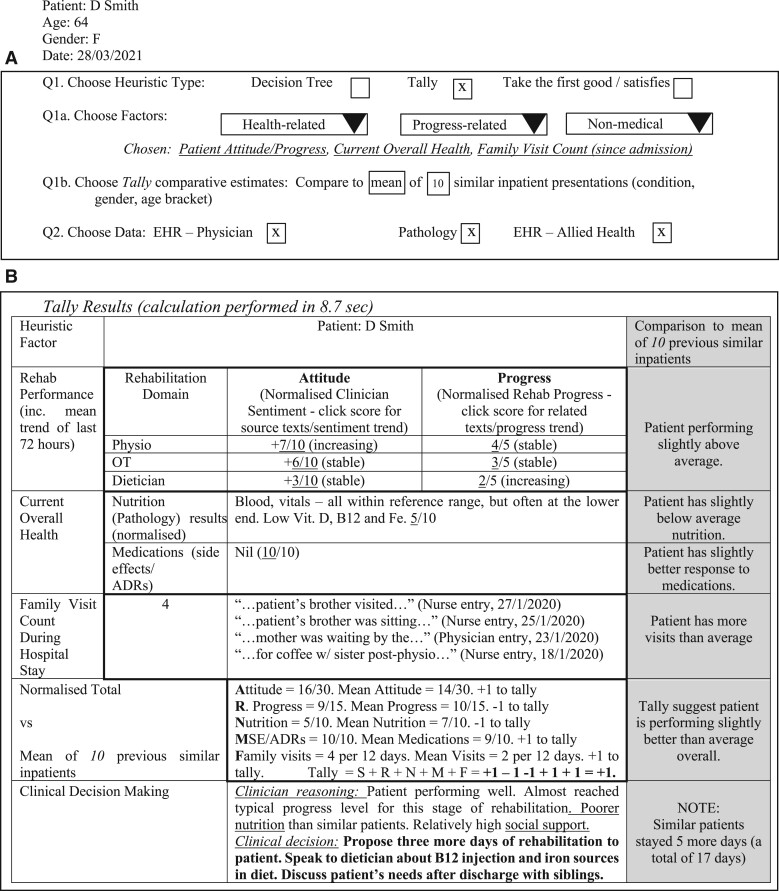
An indicative CDSS relevant to a hospital discharge decision scenario. (**A**) The treating clinician flexibly selects the heuristic form (in this case, a tally), with context and task-relevant inputs and data sources, and a relevant comparative baseline. (**B**) The platform then produces and tallies the scores for all heuristic factors, leveraging NLP, helping the clinician to determine a suitable course of action.

In Figure 2, we illustrate a sample CDSS tool based on this scenario that aims to help clinicians manage a discharge decision for a chronic heart failure patient in a rehabilitation hospital. The clinician explicitly visualizes a clinical heuristic (here, a tally heuristic but other approaches may better suit specific clinical contexts) to answer “How” and “Why” questions such as those posed above. Tally heuristics have been found to be both fast and effective in complex clinical situations when compared to more complex prediction tools.^40^^,^^70^ The tally heuristic categorizes factors for and against a given clinical decision and then tallies them together to help the clinician determine a course of action. In this hypothetical tool, the system determines whether the patient is performing better or worse than the average patient over a set of typical benchmarks. The cNLP system could then support this by searching the clinical narrative for each evidence point (ie, each “What” and “How many” question, such as number of social visits, patient progress, and health). The clinician can then make an informed patient care decision.

The tally heuristic used in this example is admittedly very simple. This approach does not preclude more sophisticated modeling, including statistical or predictive modeling that makes use of the same (or additional) variables that are surfaced in the CDSS system. Indeed, data visualization methods could be used to support clinician exploration of surfaced patient characteristics in the record in a comparative or correlative manner, or statistical weighting could be added to make the CDSS more robust.

The key points here are to identify the factors that are relevant to the clinical decision, to surface them from the notes or other patient data (using cNLP as required), and to present them concisely, providing the information that a clinician needs to make a decision. In short, we have transformed an end-to-end readmission prediction tool into a clinical tool supporting a discharge decision, and shifted the use of NLP as a feature extractor or end-to-end recommendation model in a fully automated tool to targeted evidence gathering, supporting human decisions. We emphasize that this is only a sketch of the concept; codesign with health care professionals is required to determine how such data-supported decision-making technologies can best support specific usage contexts, including identifying specific information needs and defining scenarios of use.^71^

In fact, early examples of cNLP systems exist that illustrated this approach, including the use of cNLP to populate a “Structured Narrative Database” consisting of specific fields extracted from clinical texts to support clinical audits,^72^ or to enable identification of infectious patients or establish the need for inhaled anti-inflammatory agents in asthma patients^73^ utilizing cNLP to encode reports.^24^ The methods utilized by these systems were perhaps overly simplistic (eg, the highly literal rule [*If strings “normal,” “good,” or “clear” occur BEFORE term “breath sounds,” score = 0*] from^73^) and they were not typically designed for active clinical use due to lack of appropriate electronic health record systems^23^ or a need for retrospective surveillance or quality assurance. Nevertheless, they prioritized collaboration between medical practitioners and informaticians in defining fine-grained concepts relevant to a given clinical scenario. As methods have increased in sophistication, our sights have shifted from improving findability and organization of information to full automation of decision-making. Perhaps it is time to revisit these earlier scenarios.

## DISCUSSION

Our proposal is in line with recent commentary from thought leaders in AI in Medicine. Eric Topol and colleagues have observed that “deployment of medical AI systems in routine clinical care presents an important yet largely unfulfilled opportunity.”^74^ They cite not only the need for systems that leverage multiple sources of medical data, including clinical texts, but also the need for human-in-the-loop setups that consider how AI can assist decision-making most effectively. While we focus here on cNLP, much of what we say is also applicable to other AI or machine learning systems. We single out cNLP for discussion here because of the clear opportunity in the context of medical texts to define (sub)tasks that enable alignment between the information that is needed for clinical decisions, and the information targeted by cNLP. In particular, the terms and concepts that constitute clinical texts, and the relationships between them, may correspond to precisely the patient details needed to assist clinical decision-making.

There are several key recognized issues that constrain the clinical utility of cNLP that merit discussion in the context of our proposal. These are elaborated below:

### Issue 1: under-performance of cNLP systems on complex language processing tasks

One key reason for the limited cNLP deployment is that the NLP platforms developed so far have fundamental limitations that impede their comprehension of real-world clinical data. Clinical NLP pipelines generally struggle with linguistic issues such as word or phrase ambiguity, complex semantic roles (eg, differentiating subject and object), temporality of events, or information that a clinician would intuitively flag as “missing” and fill in implicitly (eg, assumption of a positive characteristic due to a lack of documentation of the *absence* of that feature^75^). Furthermore, more basic challenges such as word misspellings or significant variations between language used across organizations impact performance.^12^^,^^20^ It has also been observed that many modern NLP systems are “like a mouth without a brain”^76^ or “stochastic parrots”^77^—most particularly evident in applications such as medical report generation, where reports have been optimized to “*look real* rather than to *predict right*”^78^ and are biased toward normal findings.^79^ cNLP systems will need to somehow incorporate a certain degree of pragmatism or “common sense” before the aforementioned issues can be fixed. Therefore, the linguistic analysis limitations and lack of pragmatic intelligence of cNLP—commonly referred to as “weak AI”^80^—limit its deployment within complex healthcare environments. Adopting an approach that focuses on more achievable NLP tasks with explicit relevance to a specific clinical decision task may mitigate against this problem.

### Issue 2: simplification of real-world problems

A related concern for cNLP is its simplification of real-world clinical problems. NLP model pipelines generally simplify a real-world problem into a linear “goal—task—solution” model; that is, a “task as decision” approach. As discussed above, cNLP systems are usually in line with the NLP tasks of information extraction, involving migrating unstructured data to a standardized form,^8^^,^^81^ or classification of texts into categories such as for disease case detection.^82^^,^^83^ While cNLP platforms (whether independent, or as components of CDSS) do not need to “understand” a task as clinicians do, they would need deeper sophistication to provide useful recommendations, predictions, or clinical workflow improvements.^20^ Furthermore, simplification (at best) leads to an NLP task and outcome that comprises 1 or 2 components of the many clinical tasks involved in patient care. Thus, cNLP is typically used for a select set of simplified “what” questions, but not the more complex, and clinically useful “how” or “why” questions.^59^ Additionally, cNLP models usually produce binary outputs (presence/absence, true/false) for one or more medical variables often without considering how they meaningfully associate with other medical conditions or events. Several reviews of clinical information extraction applications have found that the vast majority of cNLP models involved an attempt to automatically detect the presence or absence of a disease or injury, adverse medical or treatment events, or patient characteristics, with a small proportion also extracting numeric values from narrative text.^1^^,^^84^^,^^85^ These lend themselves to “needs,” better than “decisions,” and may find more relevance in a decision support context.

### Issue 3: explainability

What we are proposing is distinct from the current focus in the artificial intelligence community on explainability and interpretability of sophisticated *black box* statistical or machine learning-based models.^86^ As Holzinger and colleagues have put it, “Explainability is at least as old as AI itself and rather a problem that has been caused by it.”^87^ While we wholeheartedly support efforts to make AI model decisions explainable, here, we instead suggest that not every decision is suited directly to AI. Perhaps by more carefully scoping the tasks we demand of our AI, and directly engaging powerful human intelligence capabilities, we can both arrive at more effective clinical decisions and avoid the need to immediately solve the problem of explainability.

## CONCLUSION: MOVING FROM PROBLEMS TO SOLUTIONS

We have provided an indicative example that illustrates one of many possible ways to integrate NLP into existing clinical decision-making. This system could improve clinician trust by meeting clinician needs and complementing their judgment, and such systems may be a good starting point for future cNLP deployments.

Researchers need to work closely with clinicians to explicitly and flexibly incorporate and operationalize their needs into CDSS or other systems, so that cNLP tools can usefully contribute to decisions in partnership with clinicians. This allows for a shift from the “task as decision” model to a “task as need” model. Further codesign with researchers in other areas including implementation science, CDSS, user experience and clinical decision analysis would also support development of more meaningful cNLP systems.^88^^,^^89^

The “tasks as needs” model represents a paradigm shift in cNLP to focus on *supporting* clinicians rather than *emulating* clinicians, and to bring cNLP in line with Friedman’s Fundamental Theorem. Under this approach, cNLP researchers will produce systems that more effectively integrate into clinical workflows and facilitate a closer working relationship between clinicians and their decision-support tools. It is in this way that cNLP researchers can realize immediate clinical gains and can increase the likelihood that clinicians will benefit from adopting cNLP systems.

## FUNDING

This work was supported by the Australian National Health and Medical Research Council (NHMRC) Centre for Research Excellence in Digital Health (CREDiH), grant number APP1134919, to author KV.

## AUTHOR CONTRIBUTIONS

KV and RL conceptualized the scope of the paper and planned the study. AL conducted detailed literature review, synthesized the material, defined the case study, and drafted the manuscript. All authors contributed to critically revising the manuscript. KV led the additions and revisions that resulted from the review process. All authors approved the final version to be published.
